# Supplementary material for: Direct Photochemical Synthesis of Substituted Benzo[b]fluorenes
Source: Org Lett. 2024 Nov 25;26(48):10364–8. doi: 10.1021/acs.orglett.4c03978 (PMC11629382; doi:10.1021/acs.orglett.4c03978)
Supplement: Supplementary file 2 — ol4c03978_si_002.zip [file ol4c03978_si_002.zip › FID for Publication/5s/20240724_RC-2-C-5-132A_01/plots/RC-2-C-5-132A_CARBON_20240724_01_plot01.pdf]

RC-2-C-5-132A

Location	**4**	Pulse sequence	**CARBON**	Temperature	**25**	Suboperator	**RuairiCrawford**
Date collected	**2024-07-24**	Solvent	**acetone**	Spectrometer	**localhost.localdomain-vnmrs500**	Operator	**MBaumann**

SAMPLE

date	**Jul 24 2024**
solvent	**acetone**
file	**/home/data/MBaumann/cscb500/RuairiCrawford/2024/20240724_RC-2-C-5-132A_01/RC-2-C-5-132A_CARBON_20240724_01.fid**

ACQUISITION

sw	**31250.0**
at	**1.049**
np	**65536**
fb	**17000**
bs	**8**
ss	**128**
d1	**1.000**
nt	**2000**
ct	**2000**

TRANSMITTER

tn	**C13**
sfrq	**125.669**
tof	**1913.4**
tpwr	**58**
pw	**7.700**

DECOUPLER

dn	**H1**
dof	**0**
dm	**yyy**
decwave	**w**
dpwr	**36**
dmf	**13423**

PRESATURATION

satmode	**n**
wet	**n**

SPECIAL

temp	**25.0**
gain	**50**
spin	**0**
hst	**0.008**
pw90	**15.400**
alfa	**10.000**

FLAGS

il	**n**
in	**n**
dp	**y**
hs	**nn**

PROCESSING

lb	**0.50**
fn	**not used**

DISPLAY

sp	**-1802.9**
wp	**31250.0**
rfl	**1802.9**
rfp	**0**
rp	**-125.5**
lp	**0**

PLOT

wc	**169**
sc	**8**
vs	**3021**
th	**3**
ai cdc ph	

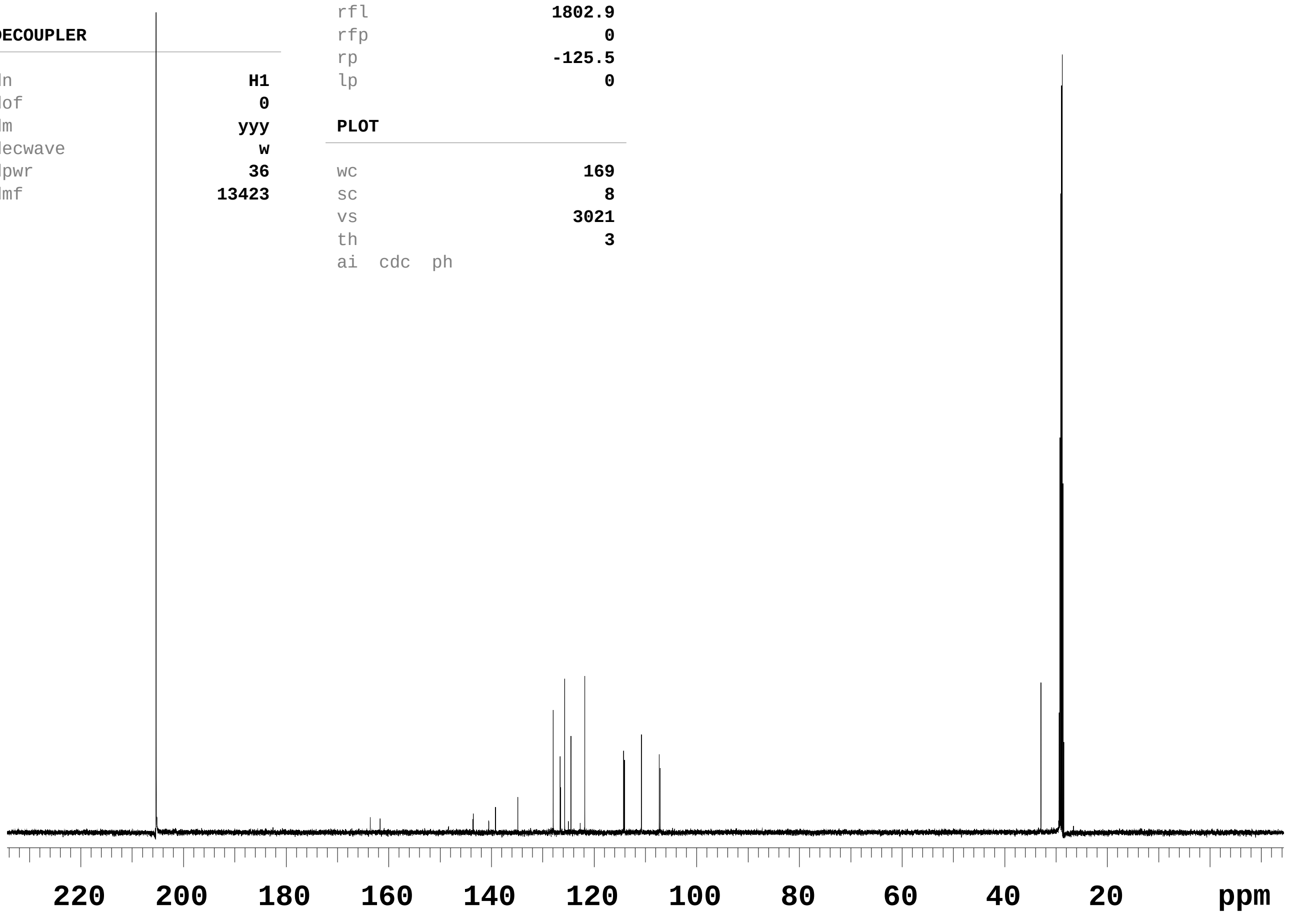

Plotname: RC-2-C-5-132A_CARBON_20240724_01_plot01

Data file /home/data/MBaumann/cscb500/RuairiCrawford/2024/20240724_RC-2-C-5-132A_01/RC-2-C-5-132A_CARBON_20240724_01.fid Plot date 2024-07-24
